# Leonardo di ser piero da Vinci–a bridge between ancient and post-Galenic anatomy based on studies of the ventricular system of the brain

**DOI:** 10.3389/fnana.2026.1899378

**Published:** 2026-08-06

**Authors:** Jarosław Dzierżanowski, Michał Krakowiak, Julia Stelmach, Natalia Haczyk, Piotr Zieliński

**Affiliations:** 1 Department of Neurosurgery, Medical University of Gdańsk, Gdańsk, Poland; 2 Polish Scientific Circle of Neurosurgery, Medical University of Gdańsk, Gdańsk, Poland

**Keywords:** drawings of Leonardo da Vinci, history of neuroanatomy, human ventricular system, neuroanatomy, ventricular system of the brain

## Abstract

When thinking about the beginning of modern anatomical drawings that reflect topography in detail, our thoughts often turn to the more than 200 woodcuts included in Andreas Vesalius’s “De humani corporis fabrica libri septem.” However, Leonardo da Vinci also produced detailed anatomical sketches before the publication of the Fabrica, including drawings that addressed the cerebral ventricular system. The goal of this narrative historical review is to examine how medical knowledge of the ventricular system evolved from antiquity, represented by Galen of Pergamon and Hippocrates of Kos, toward post-Galenic anatomy. This analysis is based on four sketches created between approximately 1487 and 1509, held in the collections of the Royal Library at Windsor, England, and the Schlossmuseum in Weimar, Germany. Although medical literature frequently references Leonardo’s anatomical work, relatively few studies have focused specifically on the morphology of the ventricular system while also considering the physiology and philosophy of the turn of the 15th and 16th centuries. This study therefore analyzes Leonardo’s ventricular drawings as an important transitional episode in the visual history of neuroanatomy. At the same time, it acknowledges that Leonardo’s anatomical studies were largely unpublished during his lifetime, that their influence on later anatomists remains uncertain, and that retrospective interpretation of historical drawings requires caution.

## 1 Introduction

The widely accepted view states that until the publication of Andreas Vesalius’s “De humani corporis fabrica libri septem,” medical knowledge was largely rooted in the anatomy of Galen of Pergamon. This anatomical tradition incorporated theories from ancient philosophers, such as Aristotle in “De Anima,” as well as early Christian theologians like Nemesius of Emesa, whose work “De natura hominis” was embraced by the Catholic Church for its doctrinal concordance. Existing literature on the history of medicine often transitions abruptly from rudimentary concepts based on animal dissections (such as the pigs and macaques studied by Galen) to the complex systems proposed by Renaissance anatomists like Juan Valverde de Amusco and Costanzo Varolio, who relied on human dissection and empirical experimentation. This same observation applies to the ventricular system. While it was likely recognized by Hippocrates (Swanson, 2007) and further detailed by Herophilus of Alexandria (c. 335–280 BC) (Solmsen, 1961; Wiltse and Pait, 1998), the system was represented for over 1,800 years, until Vesalius’s 1543 treatise, as a series of three ventricles arranged linearly. According to conventional ancient philosophy, this system harbored the “soul and spirit” (spiritus animalis), described as a “life-giving vapor providing vigor to the body and mind” (Milhorat, 1975; Rasmussen et al., 2022). These ventricles were thought to facilitate sensation, reason, and memory, respectively. It is only in later works, such as Johannes Dryander’s “Anatomiae, hoc est, corporis humani dissectionis pars prior…” (Dryander, 1536) or the aforementioned “De humani corporis…” of Vesalius (1543), that a pyramidal arrangement is encountered: two anterior ventricles (now termed lateral) positioned most superiorly, followed by a middle ventricle (the third) and a posterior ventricle (the fourth). However, what was the conceptual bridge between the eras of Galen and Vesalius.

The goal of this study is to examine, based on Leonardo da Vinci’s series of ventricular drawings, the gradual evolution of anatomical knowledge concerning the ventricular system. How did the thought of a single individual move from inherited ancient and medieval concepts toward a more empirical anatomical approach? To what extent did the wax injection experiment allow Leonardo to visualize the spatial distribution of the ventricular system? How might these drawings have contributed to anatomical knowledge if they had been published with the substantive support of Marcantonio della Torre? Finally, How should Leonardo’s work be situated within the historical transition between Galenic, medieval, and post-Galenic anatomy?

## 2 Materials and methods

**FIGURE 1:**
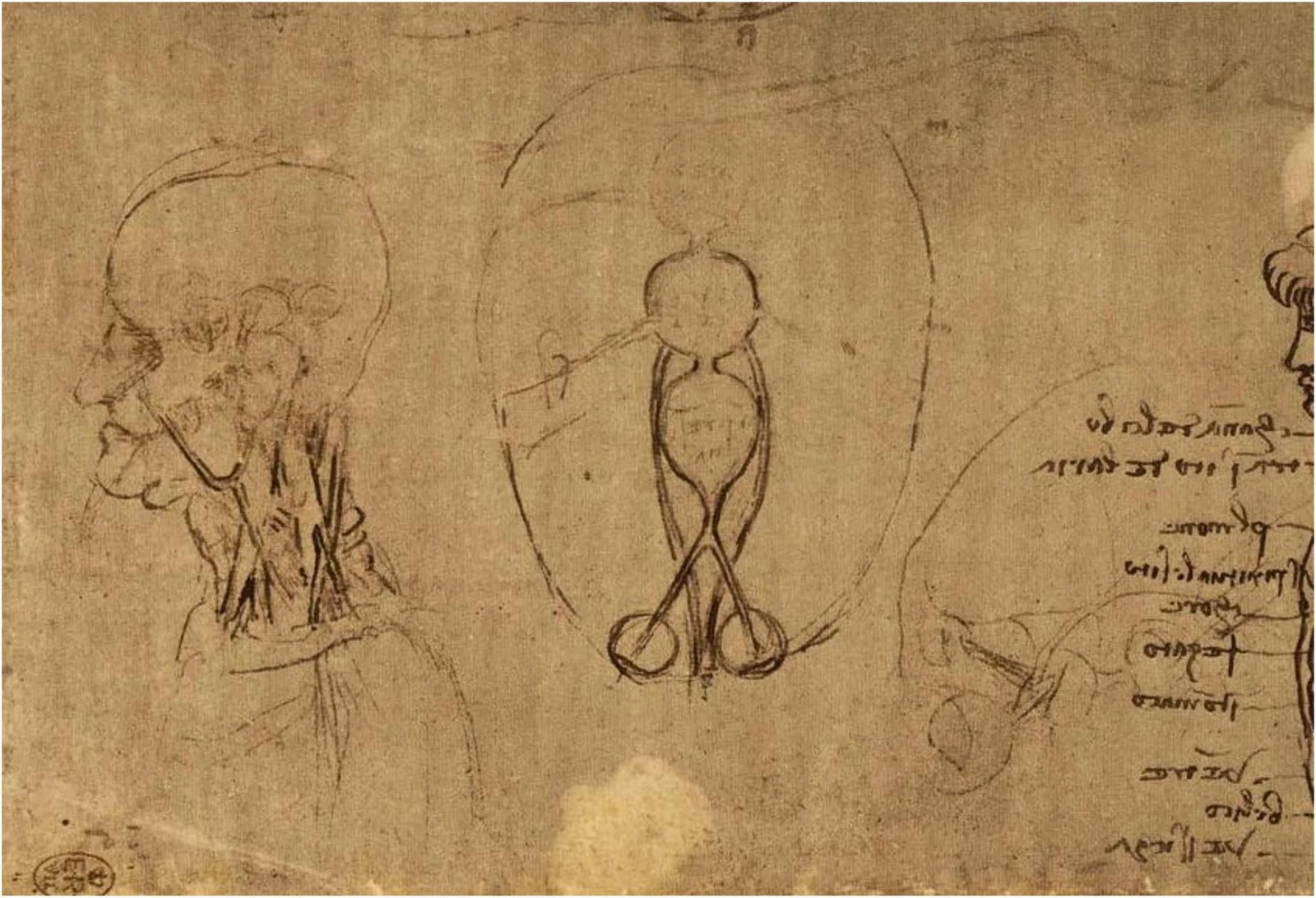
“The Cell Doctrine of Cerebral Localization.”

**FIGURE 2:**
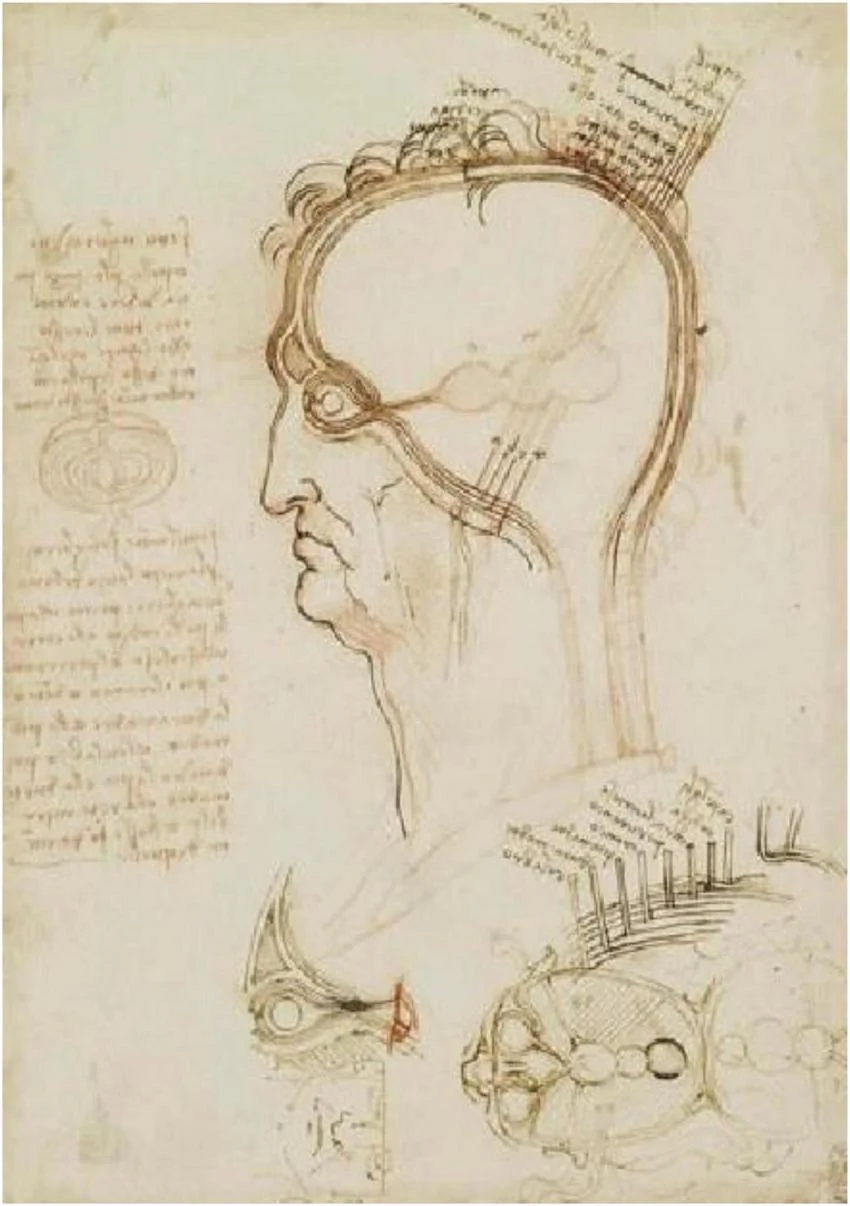
“The layers of the scalp, and the cerebral ventricles”/“Studies of the head.”

**FIGURE 3:**
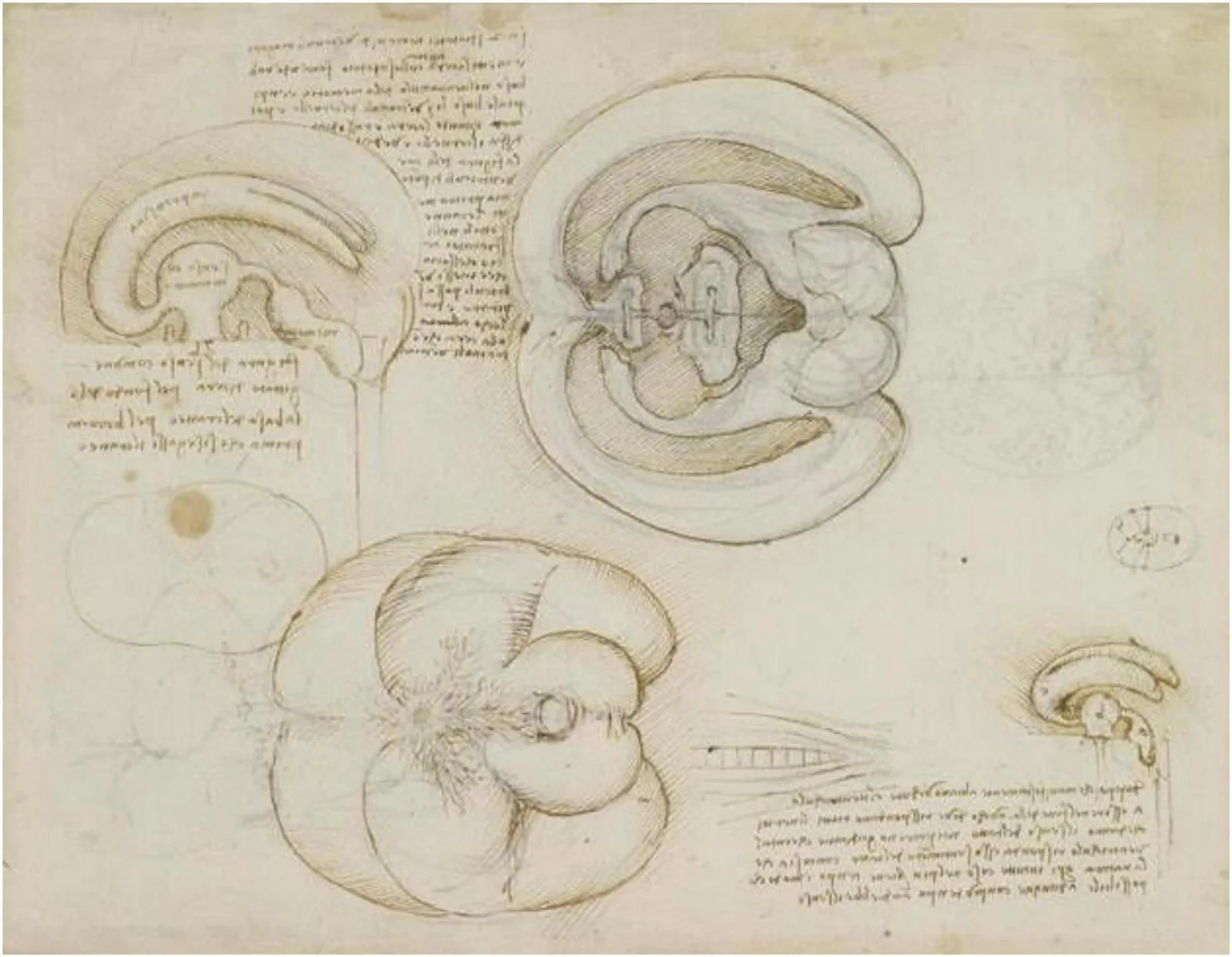
“The brain.”

**FIGURE 4:**
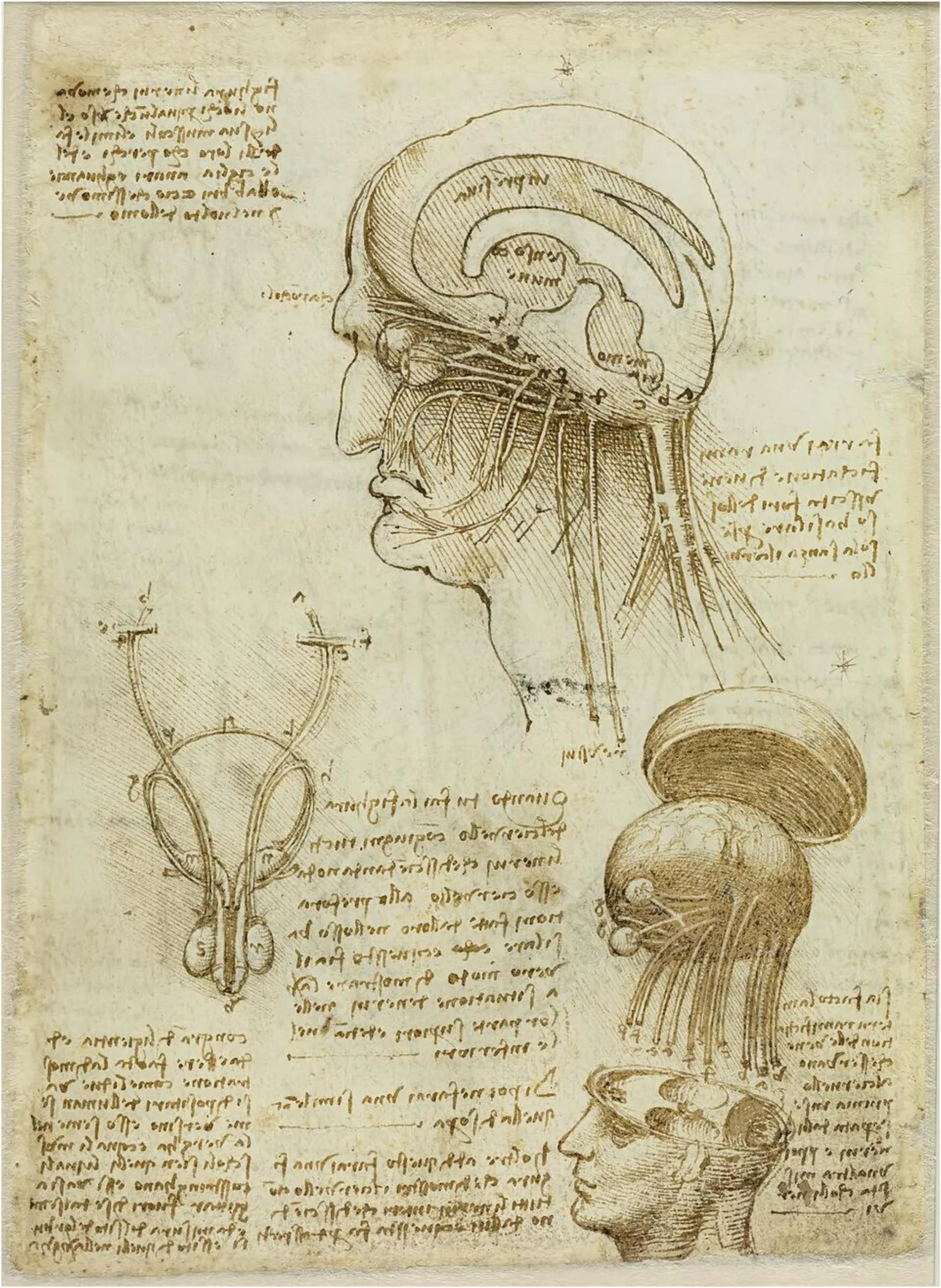
“Manuscript containing anatomical drawings”/“Weimar Folio.”

Four anatomical sketches by Leonardo da Vinci, created between approximately 1487 and 1509, were analyzed. Figures 1–3 are held in the Royal Library at Windsor, England, while Figure 4 is housed in the Schlossmuseum in Weimar, Germany. The present article is a narrative, image-based historical review rather than a systematic review or meta-analysis. The drawings were selected because they directly depict the ventricular system, represent successive stages in Leonardo’s anatomical reasoning, and are available through established catalog descriptions that allow comparison with published historical and anatomical literature. Modern terms such as lateral ventricle, third ventricle, fourth ventricle, cerebral aqueduct, cranial nerves, and cerebrospinal fluid are used descriptively for contemporary readers and should not be understood as terms used by Leonardo himself. The subject of this analysis is therefore the structure of the ventricular system in relation to the contemporary state of knowledge and how that knowledge evolved through dissections and experiments.

Figure 1 titled “The Cell Doctrine of Cerebral Lo calization” (cataloged as K/P 4r, ca. 1487), from “The Drawings of Leonardo da Vinci” in the Collection of Her Majesty the Queen at Windsor Castle (Maestro and Rolando, 2015), presents a transverse section of the head at the level of the eyeballs, showing a linear arrangement of three ventricles.

Figure 2 titled “The layers of the scalp, and the cerebral ventricles” (verso: “Studies of the head”), c. 1490–1492 (cataloged as RCIN 912603), presents both transverse and sagittal sections of the head at the level of the eyeballs, depicting the triple-ventricular arrangement, the optic chiasm, and the meningeal system.

Figure 3 titled “The brain,” c. 1508–1509 (cataloged as RCIN 919127), presents transverse and sagittal sections of the brain with a clearly visible ventricular system. This drawing is associated with the wax injection technique (injecting melted wax into the cerebral ventricles) used by Leonardo to examine the internal structure of an ox’s brain.

Figure 4 titled “Manuskript mit anatomischen Zeichnungen” (Manuscript containing anatomical drawings; commonly known as the “Weimar Folio”), created between 1506 and 1508, presents, among other features, a sagittal section of the head showing the ventricular system, the emerging cranial nerves, and the junction of the brain and spinal cord.

## 3 Discussion

Even though only dozens of Leonardo’s anatomical drawings have survived, his extensive notes, written in his signature “mirror writing,” from right to left, provide detailed insight into his anatomical interests. While studying the circulatory system, he challenged elements of traditional physiology and investigated the role of the heart, cardiac valves, and hemodynamics. Leonardo’s research extended to the genitourinary and gastrointestinal systems, including descriptions that have been discussed in relation to liver cirrhosis. He was also interested in the organs of vision, the optic pathways, the tracheal opening, osteology, the physiological curves of the human spine, the structure of the cervical vertebrae, the articulations of the axis (C2), musculature, peripheral nerves, the brachial plexus, and the ventricular system. These observations show the breadth of his anatomical work, but they should be treated as contextual background rather than proof of direct influence on later anatomy. His empirical approach to medicine and the evolution of his anatomical understanding are best exemplified, for the purposes of this study, by four anatomical plates detailing the brain’s ventricular structure.

During antiquity, the Middle Ages, and the early Renaissance, anatomical knowledge was primarily derived from ancient Greek schools. Scholars relied on treatises by Hippocrates of Kos, Herophilus of Chalcedon, Erasistratus of Ceos, and Rufus of Ephesus, specifically in his work “On the Names of the Parts of the Human Body.” The most influential figure was Galen of Pergamon (Claudius Galenus), whose works “De Usu Partium Corporis Humani” and “De Anatomicis Administrationibus” shaped anatomical thought for centuries (Clarke and O’Malley, 1996; Galen, 1968; Rocca, 2003; Solmsen, 1961; Swanson, 2007; Wiltse and Pait, 1998). To explain organ function, pathogenesis, and potential treatments, anatomy was often intertwined with philosophy. This included “humor theory” (the system of vital bodily fluids) described by Hippocrates, Alcmaeon of Croton, and Empedocles. It also incorporated concepts of the “soul” from Plato’s Timaeus or the “rational soul” (psyche) from Aristotle’s De Anima. Under this “spiritualized physiology,” different anatomical regions were believed to house specific spirits (pneuma in Greek, spiritus in Latin) responsible for health and emotions. Consequently, medieval doctrine maintained that the heart housed the “vital spirit,” governing passion and executive functions, while the liver contained the “natural” or “vegetative spirit,” responsible for desire and nutrition (Hankinson, 1991; Lloyd, 2007; Rocca, 2003; van der Eijk, 2008).

What, then, were the contents of the ventricular system, and what role did it play within the human organism? This question was addressed in the fourth century (c. 390–400 A.D.) by Bishop Nemesius of Emesa in his “Doctrine of Ventricle Localization of Mental Functioning.” His work was partly predicated on Galen’s concept of “psychic pneuma” (van der Eijk, 2008). Within this framework, the ventricles were considered the seat of the spirits responsible for reason, imagination, and memory. Furthermore, it was believed that “vital spirit” traveled to the brain via the arteries, while the “natural soul” was transported through the veins. This conceptualization of mental functioning (as spirit or soul) was adopted by the Catholic Church, further propagated by Saint Augustine of Hippo, and persisted for nearly 1,300 years despite the dynamic evolution of anatomical knowledge during the Late Middle Ages and the early Renaissance.

Leonardo da Vinci was not only an insightful observer and experimenter but also a conscientious researcher who grounded his studies in the available literature of the period. Aided by wealthy patrons, he gained access to the essential compendia of European and Arabic scholars. Leonardo also possessed a sizeable book collection, including: “De anatomia partium hominis” by Herophilus, “Anathomia Corporis Humani” by Mondino de Luzzi, “De Animalibus” by Albertus Magnus, “Chirurgia magna” by Guy de Chauliac, and the “Medicinae” by Ibn Sînâ (Avicenna), as well as Averroes’ “The General Principles of Medicine” (Pevsner, 2002). The synthesis of ventricular structure and function found in these texts is reflected in Leonardo’s early drawings: “Cell Doctrine of Cerebral Localization” (ca. 1487) and “The layers of the scalp, and the cerebral ventricles” (1490–92). These works follow the, at the time, standard patterns; therefore, the ventricles are positioned linearly along the brain’s central axis, comprising the anterior (now termed lateral), middle (now termed the third), and posterior (now termed the fourth) ventricles. It was established that partitions separated these chambers and that “tunnels,” cranial nerves, connected to the anterior ventricle. This summarized the prevailing morphological knowledge of the era. However, What did physiological knowledge look like? The anterior ventricle served as the input center for all sensory perceptions from the external environment, the “sensus communis,” integrated with imagination. These perceptions (today referred to as stimuli) were believed to be transported via the cranial nerves to this anterior chamber. Cognition and the “mind” were localized in the middle ventricle, the site where the selection, verification, and potential rejection of incoming perceptions occurred. Validated perceptions were then transferred to the posterior ventricle, which functioned as the memory center. This schematic model of cerebral physiology persisted with only minor modifications through the time of Leonardo and Marcantonio della Torre. It remained largely unchanged for decades, even following the publication of Andreas Vesalius’s De humani corporis fabrica (Tubbs et al., 2018). It appears that Thomas Willis’s 1664 work, “Cerebri anatome,” eventually superseded this simplistic neuroanatomy, establishing the foundations of modern neurology (Arráez-Aybar et al., 2015).

While Leonardo’s first two drawings do not represent the finalization of his research, certain noteworthy conceptual features are already evident within them. In the “Cell Doctrine of Cerebral Localization” (the earliest illustration in the series), Leonardo da Vinci depicts only the optic fibers as entering the anterior ventricle. The remaining senses, namely smell, taste, and hearing, are shown entering the middle ventricle directly via cochlear and olfactory fibers. Notably, the latter was not classified as a sensus stricto nerve for several centuries, spanning the post-Galenic period through the early Renaissance. This representation of a direct connection between the middle ventricle and these fibers appears difficult to reconcile with the prevailing doctrine of the time. Leonardo identified the anterior ventricle as the seat of the “imprensiva,” while the soul was localized within the middle ventricle, the senso comune, which received sensory impulses or “sentimenti” (Wysiadecki et al., 2025). Furthermore, the middle ventricle served as the cortical source of motor commands, transmitting “sentimenti” along the motor nerves to the musculoskeletal system. Returning to Figure 1, the work lacks comprehensive anatomical explanations. Given Da Vinci’s renowned meticulousness, one might argue that this was a deliberate and conscious sketch; however, the specific rationale behind these conceptual choices remains unknown.

The anatomical representation shifts significantly in Figure 2, often referred to as “Resembling an Onion,” e.g., in the book of Cianchi (2017), created 3–5 years later. Here, the anterior ventricle is directly connected with the optic and olfactory fibers, while the remaining cranial nerves are excluded from the ventricular system. The paramount importance Leonardo accorded to vision and hearing within this system is evidenced by his observation: “And where the sense that ministers to the soul is not at the service of the soul, all the functions of that sense are also wanting in that man’s life, as is seen in those born mute and blind” (Kemp, 1972). This raises the question of why Leonardo did not base these initial drawings on direct dissections, as he did in his subsequent masterpieces. The answer to his schematic treatment of the ventricular system appears to lie within the composition of Figure 2 itself. In the book “Leonardo Anatomy” written by Cianchi (2017), it was entitled “Resembling an Onion,” derived from an onion cross-section sketched on the right margin. Leonardo posits that the layers of an onion, tightly adherent and concentric, serve as an analogue for the human head: “If you will cut an onion through the middle, you will be able to see and enumerate all the coats or rinds which circularly clothe the center… Similarly, if you will cut through the middle of the head of a man, you will first cut the hairs, then the scalp, then the muscular flesh and pericranium, then the cranium; and inside, the dura mater, the pia mater, and the brain” (Pevsner, 2002). A corresponding transverse section at the level of the orbits is illustrated at the base of the sketch. Consequently, it is plausible that the ventricles were intended as a conceptual adjunct, a schematic to elucidate the layered architecture of the human body and the principles of anatomical topography. This hypothesis may also explain why both the transverse sections in Figures 1, 2, as well as the sagittal section in Figure 2, depict only a single anterior ventricle despite Leonardo’s awareness of their paired nature. This anatomical arrangement had already been described by Galen in *On Anatomical Procedures* and was subsequently reproduced by his followers throughout the medieval period, ranging from Arab and Muslim scholars to anatomists associated with the universities of Paris and Bologna. This “inconsistency” is notably absent in Figures 3, 4.

Leonardo returned to intensive anatomical studies between 1506 and 1513. He was granted permission to perform human dissections at hospitals in Florence, Milan, and Rome, which provided him with exceptional opportunities for the period. He was a well-known person in the world of art and was supported by multiple wealthy patrons, including Pope Leo Xth (Sterpetti, 2016). In literature, we can frequently find inaccurate or largely simplified information that the Catholic Church prohibited performing human dissections, or that the fact that it was the period of the Holy Inquisition somehow hindered the freedom of science. However, the papal bull, which is frequently quoted to prove that, did not prohibit the act of dissection. It prohibited desecration of bodies, especially those of pilgrims who came to Rome, and separating the bones from the body, which were then transported to their respective homelands for burial. It also allowed post-mortem examinations when there was suspicion of an infectious disease posing an epidemic threat, or when the determination of the cause of death was impossible otherwise. Leonardo’s research took on a new dimension through his collaboration (1510–1511) with Marcantonio della Torre, a professor of anatomy at the University of Pavia. Their joint efforts resulted in exquisite anatomical drawings, potentially accompanied by a co-authored descriptive text. While these were planned for publication as a medical treatise, the project was curtailed by della Torre’s death from the plague. For the subsequent 2 years, Leonardo continued sketching human organs and conducting experiments, such as those concerning the circulatory system; nevertheless, due to their lack of a systematic methodology and the absence of publication during his lifetime, these findings did not become part of formal scholarly anatomy at that time. It was not until 30 years later that the field of anatomy was transformed by Andreas Vesalius’s “De humani corporis fabrica,” which contained drawings by exceptional artists, including Johan Stephen von Kalcar and Domenico Campagnola, both pupils of Tiziano Vecelli (Titian).

Figures 3, 4 created during this period, illustrate the structure of the ventricular system. Figure 3, titled “The brain,” represents an important stage in Leonardo’s morphological studies. To render physiological dimensions and morphology more accurately, Leonardo developed a wax casting technique. His initial experiments, conducted between 1506 and 1508, utilized the heads of horses and oxen (Bayat, 2020; Schuez and Alt, 2022). In his notes, he documented the experimental procedures in detail: he inserted tubes into the ventricles and, employing self-constructed syringes, injected molten wax. Furthermore, narrow tubes served as air vents (deaerators), ensuring the wax completely and hermetically filled the ventricular system (Keele, 1964; O’Malley and Saunders, 2003; Pevsner, 2002). Subsequently, he removed the brain parenchyma to obtain a spatial model of the ventricles. As an analogy, this method is closer to anatomical casting and, in a limited sense, to the logic of corrosion casting than to modern three-dimensional printing, although Leonardo did not perform true corrosion casting because no chemical corrosion of surrounding tissue was used. The comparison with modern 3D methods should therefore be understood only as a broad conceptual parallel: both approaches seek to create a spatial model of an internal structure. Nevertheless, the conceptual approach of depicting the internal configuration of the cerebral ventricular system anticipated the ventriculography introduced by Walter E. Dandy in 1918 by over 400 years, a historical parallel discussed in detail by da Mota and Gomes (2020), Gomes Mda et al. (2015).

How do Figures 3, 4 differ from Figures 1, 2? Primarily, the arrangement of the ventricles evolved from a linear configuration (extending sequentially from the frontal to the occipital region) to a pyramidal one. The two anterior ventricles (now termed lateral ventricles) are situated in the most superior position, followed by the middle and posterior ventricles. Leonardo emphasized the direct connectivity between these cavities: the first connection between the anterior and middle ventricles, and the second between the middle and posterior ventricles. The former is not a partition, as described in the works of Ibn Sīnā and Raimondo de’Luzzi, but may be interpreted in modern terms as the interventricular foramen (the eponym “foramen of Monro” was introduced much later) (Monro, 1775). The second connection depicted is the channel leading to the fourth ventricle, which some authors have interpreted as corresponding to the cerebral aqueduct (Keele, 1964; O’Malley and Saunders, 2003). While this connection between the third and fourth ventricles was previously conceptualized as a mere opening or “window” (Leite Dos et al., 2004). Even decades after Leonardo’s wax experiments, Berengarius Carpensis, in his “Isagogaes breves,” described this structure as a “small foramen that leads to a deep vacuity in the posterior part of the middle (third) ventricle” (Leite Dos et al., 2004). However, Jacques Dubois (Jacobus Sylvius), Vesalius’s teacher (Adeloye, 1977; Leite Dos et al., 2004), described the aqueduct in his 1555 work “Isagoge” as “a long and narrow meatus…[that] passes under the corpora quadrigemina into the fourth ventricle” (Adeloye, 1977; van Gijn, 2001). Nevertheless, according to the prevailing anatomical understanding of the period, this connection was generally conceived not as a tube or aqueduct but rather as a simple opening. Leonardo’s Figures 3, 4 may be interpreted as depicting this structure in a form that more closely resembles the modern concept of the cerebral aqueduct. However, this interpretation remains controversial. In particular, Steinsiepe and Hauser (2022), using graphical analysis and MRI-based reconstructions, have argued that such a reading results from a misinterpretation of an unusual section plane and the specific mode of representation employed by Leonardo. Their study illustrates how modern radiological and anatomical analyses can challenge long-standing assumptions, transforming what once appeared to be a settled interpretation into an ongoing subject of scholarly debate.

Figure 3, notwithstanding its originality at the time, exhibits certain structural limitations. These stem primarily from the inherent limitations of wax casting methodologies. While the anterior ventricles are clearly demarcated by the frontal and occipital horns (although these were not formally named at the time), the temporal horns are notably absent. This omission is consistent with the rapid solidification of wax, which limits its distribution into the most distal and inferior ventricular regions. The temporal horns are characterized by an inferior position and prominent curvature, making them difficult to fill. Furthermore, the experimental apparatus, comprising a system of tubes and deaerators, did not account for these specific structures. Any attempt to inject wax under excessively high pressure would have compromised the integrity of the brain parenchyma rather than ensuring the complete filling of the temporal horns. Similar morphological distortions are evident in the posterior ventricle. In the coronal plane, it lacks the characteristic rhomboidal shape, and in the sagittal plane, it fails to exhibit its typical triangular shape. Instead, it appears as an irregular structure. Moreover, compared to the other ventricles, its volume seems disproportionately large. Two explanations may account for this observation. First, it may reflect interspecies differences in the morphology of the hindbrain, particularly the fourth ventricle, between humans and large ruminants such as the ox, as described in classical veterinary anatomy textbooks (Getty, 1975). Second, it may represent an artifact resulting from the wax-injection technique itself, specifically the application of substantial injection pressure combined with an inadequate mechanism for relieving excess pressure during the procedure. An interesting perspective is introduced by Clayton and Philo, who noted that the spinal cord in Leonardo’s drawings is not oriented horizontally, as would be expected in most quadrupeds, and that the cerebellum “has a human position and relative size” (Clayton and Philo, 2011; Steinsiepe and Hauser, 2022). As they observed, “the spinal cord in the drawings is not directed horizontally as it is in most quadrupeds, and that the cerebellum is human in position and relative size” (Clayton and Philo, 2011; Steinsiepe and Hauser, 2022). These observations raise a fundamental question regarding the anatomical identity of the specimen represented in Leonardo’s drawings.

Another unresolved piece of the anatomical-historical puzzle regarding Leonardo’s ventricular drawings is the omission of the choroid plexus and cerebrospinal fluid (CSF). These entities were recognized in antiquity, though their physiological roles remained elusive. The earliest description of the choroid plexus (referred to as the “chorioid meninx”) likely originates from Herophilos of Chalcedon (335–280 BC) and Rufus of Ephesus (c. 100 AD), the latter of whom employed the term “chorioid tunic” to describe both the ependyma and the choroid plexus (Mortazavi et al., 2014). While the choroid plexus is depicted in Vesalius’s “De humani corporis fabrica” (Vesalius, 1543), it was not until 1854 that Ernest Faivre formally linked the structure with the site of CSF production (MacAulay et al., 2022; Rasmussen et al., 2022; Sooke, 2018). The earliest record of CSF is found in the Edwin Smith Papyrus (c. 1500 BC), which is believed to be a copy of a significantly older document dating back to 3000–2500 BC (Standring, 2016). This papyrus describes fluid leakage secondary to a comminuted skull fracture. Later, Hippocrates of Kos (460–370 BC), the “Father of Medicine,” referred to the CSF simply as “water surrounding the brain” (Zhang et al., 2024).

Observations of fluid leakage following cranial fractures were documented by the Italian surgeon Roger Frugardi as early as 1170 in “Practica Chirurgica.” Similar phenomena were noted in 1518 by Berengario da Carpi, a professor of anatomy and surgery at the University of Bologna, in his treatise “Tractatus de fractura calvae sive cranei” (Parent, 2019). However, these accounts likely described fluid originating from the subarachnoid spaces rather than the ventricular system. It was only in his subsequent work, “Commentaria super anatomiam Mundini” (1521), that Berengario postulated: “In my opinion, the gross superfluidity of the brain can be seen in the majority of heads dissected because some of it is always in the ventricles of the brain” (Parent, 2019). The absence of any mention of the fluid now termed cerebrospinal fluid (CSF) in Da Vinci’s records, even during his wax casting experiments, may be attributed to several factors. First, Leonardo was not a physician and may have been unfamiliar with the contemporary surgical observations of Frugardi or Berengario. Second, as previously established, his studies were heavily influenced by Galenic doctrine, which characterized the ventricular content as a “vaporous humor,” a gaseous spirit providing vitality to the body, rather than a liquid (Herbowski, 2013). Third, Leonardo operated under significant temporal constraints. Unlike modern researchers who utilize cold storage and chemical preservatives to maintain tissue integrity, 16th-century anatomists in the Mediterranean climate were forced to conduct dissections rapidly to avoid irreversible decomposition of substrates. However, the most compelling explanation was provided by Domenico Cotugno in his 1764 work “De Ischiade Nervosa Commentarius.” Cotugno observed that Leonardo typically worked on heads severed from the torso, a procedure that inevitably caused the CSF to drain through the foramen magnum (Di Ieva and Yasargil, 2008). Given that the total ventricular CSF volume is relatively small, with the fourth ventricle containing a mere 1 ml and the entire system totaling approximately 20 ml, such leakage would leave the cavities seemingly empty. Leonardo’s omission should therefore be interpreted cautiously and in the context of the limited physiological understanding of CSF in the early 16th century.

Figure 4 (“Manuscript containing anatomical drawings”) from the Weimar collection was created between 1506 and 1508, predating “The Shape of the Brain” by 2 years. However, it is analyzed last in this study for a methodological reason. This drawing may represent a step away from the perceived direct connection between the cranial nerves and the anterior ventricle. In this illustration, one can trace the olfactory, optic, trigeminal, cochlear, facial, and vagus nerves (this nomenclature was not yet established), none of which connect to the ventricular system. In the lower section of Figure 4, all these nerves emerge from the base of the brain through cranial foramina. This raises the question: What became of the theory that perceptions (imprensiva) were delivered to the anterior ventricle? In practice, the concept remained largely unchanged for several decades. The Doctrine of Mental Functioning continued to be recognized by numerous scholars until the 17th century (Pevsner, 2002). Furthermore, there is no extant data to confirm whether the author addressed this discrepancy in his notes or verified his previous beliefs. Leonardo da Vinci never utilized specific anatomical names for the subdivisions of the ventricular system. Instead, he adhered to a traditional functional classification, which he detailed in Drawings 3 and 4: “impressiva” for the anterior ventricles, “senso comune” for the middle, and “memoria” for the posterior ventricle.

Leonardo’s drawings are not merely artistic masterpieces. They are carefully observed anatomical visualizations that, in some respects, depart from inherited schematic models. The depicted details may indicate advancements in medical knowledge, but progress was not always explicitly documented in his notes. He may have been unaware of the later significance that some of these observations would acquire. For instance, the cranial sinus depicted in one of his drawings has been compared with a structure later attributed to the British surgeon Nathaniel Highmore in 1651, nearly 145 years later (Schuez and Alt, 2022). Such observations are valuable, but they should be presented as historiographical interpretations rather than as definitive proof of influence or priority.

To date, only 18 double-sided pages of Leonardo’s anatomical drawings and notes have survived. These pages contain 240 figures covering the skeletal, muscular, nervous, circulatory, urinary, digestive, and reproductive systems (Sooke, 2018; Tubbs et al., 2018). The extent of the material lost irreversibly remains a subject of debate. Current estimates suggest his legacy may have originally comprised up to 8,000 pages of drawings (Sterpetti, 2016). The proportion of these dedicated to medicine and their potential subject matter remain speculative. Unfortunately, his heir, Francesco Melzi, was unable to adequately preserve this material (Tubbs et al., 2018), and the vast majority of the drawings were subsequently dispersed. Only a minor portion was published in Leonardo’s “Trattato della pittura” in France in 1651 (Keele, 1964). Because the anatomical sheets were not published during Leonardo’s lifetime, any direct influence on Vesalius, Varolio, or later anatomists remains difficult to demonstrate. Today, we can only hypothesize how they might have influenced anatomical teaching and the direction of study for contemporary anatomists had the work on human anatomy been published alongside the physician Marcantonio della Torre.

## 4 Conclusion

The comparative analysis of Leonardo da Vinci’s drawings presented here offers a focused examination of ventricular system morphology at the turn of the 15th and 16th centuries, integrating the physiological and philosophical beliefs of the era. Unlike many broader accounts of Renaissance anatomy, these sketches allow us to trace changes in Leonardo’s own representation of the ventricular system: from a more schematic, doctrine-based arrangement to a more spatial depiction informed by dissection and wax injection experiments. Although his scientific foundation was rooted in prevailing authorities, such as Galen and various Arabic anatomists, Leonardo progressively tested inherited models through observation and experiment. In this sense, he may be understood as an important transitional figure in the history of neuroanatomical visualization. His wax-injection experiments are better compared with anatomical casting approaches, rather than directly with modern three-dimensional printing, although both aim to create a spatial representation of internal structures. The significance of Leonardo’s work must be balanced against several limitations: many studies remained unpublished, some observations were based on animal material, direct influence on subsequent anatomists cannot be proven, and modern anatomical interpretation cannot be equated with Leonardo’s own conceptual framework. Nevertheless, his drawings and experiments remain a valuable source for understanding the gradual transition from ancient and medieval ventricular doctrine toward post-Galenic anatomical observation.
